# Beyond Broca’s and Wernicke’s: Functional Mapping of Ancillary Language Centers Prior to Brain Tumor Surgery

**DOI:** 10.3390/tomography9040100

**Published:** 2023-06-25

**Authors:** Ashley Lawrence, Michael Carvajal, Jacob Ormsby

**Affiliations:** 1 Center for Neuropsychological Services, University of New Mexico, MSC 10 5530 1 University of New Mexico, Albuquerque, NM 87131-5001, USA; aslawrence@salud.unm.edu (A.L.); mcarvajal@salud.unm.edu (M.C.); 2 Department of Radiology, University of New Mexico, MSC 10 5530 1 University of New Mexico, Albuquerque, NM 87131-5001, USA

**Keywords:** fMRI, Broca’s area, Wernicke’s area, language laterality, intraoperative mapping

## Abstract

Functional MRI is a well-established tool used for pre-surgical planning to help the neurosurgeon have a roadmap of critical functional areas that should be avoided, if possible, during surgery to minimize morbidity for patients with brain tumors (though this also has applications for surgical resection of epileptogenic tissue and vascular lesions). This article reviews the locations of secondary language centers within the brain along with imaging findings to help improve our confidence in our knowledge on language lateralization. Brief overviews of these language centers and their contributions to the language networks will be discussed. These language centers include primary language centers of “Broca’s Area” and “Wernicke’s Area”. However, there are multiple secondary language centers such as the dorsal lateral prefrontal cortex (DLPFC), frontal eye fields, pre- supplemental motor area (pre-SMA), Basal Temporal Language Area (BTLA), along with other areas of activation. Knowing these foci helps to increase self-assurance when discussing the nature of laterality with the neurosurgeon. By knowing secondary language centers for language lateralization, via fMRI, one can feel confident on providing neurosurgeon colleagues with appropriate information on the laterality of language in preparation for surgery.

## 1. Introduction

A maximal safe resection is the recommended first line treatment for gliomas. Awake craniotomies may result in reduced postoperative neurological deficits, due to the improved identification of functional eloquent cortices in order to better guide surgical resection [1]. A number of reviews have found that awake craniotomy with electrocortical language mapping produces fewer adverse neurological outcomes with the greater extent of the resection as well as shorter hospital stays when compared to tumor resections in eloquent areas under general anesthesia [2,3,4,5,6,7]. Awake craniotomy task selection and establishing the patient’s preoperative cognitive baseline on these tasks are essential for ensuring the reliable mapping of function and for avoiding postoperative functional deficits. The fast-paced environment of the operating room requires strategically planned mapping and monitoring tasks which are sensitive to potential deficits for each eloquent region. Intraindividual differences in functional organization (a strength of intraoperative mapping) makes the preoperative identification of language centers an essential tool for preoperative planning and task selection [8]. fMRI is a noninvasive technique that provides both the localization and lateralization of the language eloquent cortex. The technical aspects of presurgical fMRI have been discussed elsewhere (for review see Jalilianhasanpour et al., 2021) [9]. This method can be reliably used for language center identification, which can determine a patient’s language laterality as well as estimate the functional risk and inform awake craniotomy language mapping, by determining the distance of the lesion from the eloquent cortex. Additionally, preoperative fMRI is essential for mapping function and guiding the surgical approach in cases where awake craniotomy and direct electrocortical mapping is not feasible due to limited resources (e.g., limited operating room availability for the longer awake procedures), patient ineligibility for the awake procedure (e.g., psychiatric/behavioral concerns, cognitive limitations that preclude participation), or adverse intraoperative events (e.g., excessive somnolence, intraoperative seizures). In this review, we will discuss various fMRI metrics used to predict functional risk and provide an overview of key cortical regions in the language network, their contributions to language function, and methods of identifying them on presurgical fMRI.

## 2. Materials and Methods

As a review article we reviewed current literature on language activation areas within the brain along with providing our own working knowledge of these areas and their importance in presurgical mapping for brain tumor patients.

## 3. Discussion

### 3.1. Language Laterality

The traditional method for calculating a language laterality index (LI) is a subtraction of the total number of activated voxels in the right hemisphere from the total number of activated voxels in the left hemisphere, divided by the total number of activated voxels in both hemispheres (LI = (nVxL − nVxR)/(nVxL + nVxR)). This results in a value ranging from −1 to 1 in which a negative number indicates a relatively greater proportion of activated voxels in the right hemisphere and a positive number indicates a relatively greater proportion of activated voxels in the left hemisphere. Voxels in the primary visual cortex, presumed not to reflect language processing per se, are typically masked out or excluded from the LI calculation. Doing so has been shown to increase concordance between fMRI estimates of language laterality and the Wada procedure [10]. As discussed in several other reviews and empirical articles [11,12,13,14,15,16], the limitation of this method is that it depends heavily on the task and threshold of the activation selected, with higher thresholds generally resulting in greater laterality to the left hemisphere. Various alternatives have been proposed including using a fixed number of voxels to calculate the LI rather than an activation threshold [17], calculating the LI as a function of the total number of activated voxels to produce an LI curve compared to a healthy control group [18], calculating LI over a range of thresholds and creating a global LI by averaging these values [19], and creating a weighted histogram of voxel counts against threshold [20]. Brumer, De Vita, Ashmore, Jarosz, and Borri (2020) compared these aforementioned methods of LI calculation, in addition to their own proposed method [21]. In their newly proposed method, the authors set every t-value present within a left hemisphere and a right hemisphere region of interest as a threshold and recorded the number of voxels with a value above that threshold. This resulted in cumulative histograms of the obtained number of voxels by the threshold value for the left and right hemispheres. The area under these obtained curves were then subtracted; the right hemisphere from the left, divided by the total area under both curves together. In comparing these various methods of calculating LIs, Brumer and colleagues (2020) demonstrated that the traditional LI is unstable over a range of thresholds, resulting in a wide variation in the lateralization, based on the threshold chosen. This finding was also more prominent for picture naming than for verb generation or word fluency, suggesting greater interindividual variability in the LI reliability for this task. The authors reported a strong agreement among alternative LI calculation methods that account for the activation threshold of these brain regions, but recommended their newly proposed area under the curve method as it is independent of a significance threshold, stable over multiple calculations, allows comparison between subjects, and relatively easy and quick to implement considering the demands of the clinical environment. Interpretation of language LIs requires careful consideration of the method used in the calculation as well as consideration of potential lesion characteristics that may impact the BOLD signal, as well as patient factors including age and handedness.

As discussed above, use of a single statistical threshold for the calculation of the LI may bias the interpretation of laterality and has been implicated in findings of pseudo-reorganization in tumor cases. Specifically, as lower thresholds are used to detect activation, more nondominant hemisphere voxels tend to become statistically significant, resulting in a less strongly lateralized index [14]. Calculating laterality for individuals with brain tumors presents a particular challenge due to the potential that the lesion itself disrupts the local BOLD response due to a variety of factors including vascular cooption, vasogenic edema, and hemorrhage associated with the lesion [22,23]. Changes to cerebrovascular reactivity surrounding gliomas are present in both low and high grade lesions [24]. This neurovascular uncoupling could result in an incorrect language lateralization, simply because a lesion in the dominant hemisphere has sufficiently disrupted the BOLD response to render nondominant hemisphere activations proportionally greater. Therefore, contralesional activation in the setting of a tumor located in the language eloquent cortex cannot be assumed to represent an absence of risk to language function with surgical resection of the lesion [25]. In addition, neurovascular uncoupling increases the likelihood of false negative errors in the detection of perilesional eloquence due to disruptions in regional cerebral blood flow and thus the correlation between cerebral blood flow and task associated neuronal activity [26]. Hypercapnic protocols such as CO_2_ administration and breath hold tasks have been used to quantify regional variations in cerebrovascular reactivity as an adjunct to task-based fMRI to map regions where lesion associated neurovascular uncoupling may be contributing to false negative errors [27].

Task selection and ROI selection may also play an important role in the reliability of LIs in the setting of tumor. As mentioned previously, differences in the tasks used in each study impacts the interpretation of language laterality and the most reliable and robust laterality interpretation takes into account several different tasks and their nodes of overlap [28]. Pillai and colleagues compared the utility of clinical fMRI activations for language tasks previously shown to have a high concordance with the Wada procedure and intraoperative electrocortical stimulation: object naming, passive listening comprehension, reading comprehension, passive listening, silent word generation, and rhyming [29]. The authors found that rhyming and word generation provided consistent hemispheric language lateralization regardless of the tumor location in the language dominant hemisphere. Furthermore, LIs may be more reliable when calculated accounting for the tumor location. Polczynska and colleagues (2021) found that LIs were most robust when calculated using anterior ROIs (e.g., Broca’s area) except when patients had tumors in left anterior regions [25]. LIs based on posterior language sites (e.g., Wernicke’s area) were most robust for patients with anterior tumors.

Patient factors also play a significant role in the interpretation of LIs. One of the most widely used predictors of language laterality is handedness, typically assessed using the Edinburgh Handedness Inventory [30]. A majority of people are left hemisphere dominant for language (approximately 82% [31]), with a slightly greater proportion of left handed than right handed people showing right hemisphere dominance for language [32,33]. Approximately 95% of the right-handed and 70 to 80% of left-handed individuals show left hemispheric dominance for language [34]. Language laterality also changes over the lifespan, with language laterality increasing with age in childhood and adolescence [34] and decreasing in older age [35]. Certain conditions increase the risk for atypical language organization including epilepsy (particularly with onset prior to age 6) [36], schizophrenia [37], dyslexia [38], autism spectrum disorder [39], and other neurodevelopmental disorders [40]. While initial screening for conditions that increase the risk of atypical language lateralization may be useful in giving context to LIs, it should be noted that these conditions only increase the likelihood of bilateral or right hemisphere language dominance. Individuals without these conditions may still display bilateral or atypical language organization and LIs should therefore not be negated in the context of bilateral or atypical organization for right-handed people without these risk factors.

With the above, a background of tools is provided for fMRI and gaining confidence with determining language lateralization. As mentioned above, individual tasks have varying reliability for certain areas of the language network and careful consideration should be made when choosing tasks. With our institution, though, we have decided to always perform the same three language tasks of sentence completion, word generation, and object naming. The reason for this being is that it ensures standardization between operators, covers a large area of the complete language network, and allows for an overlap of tasks for further confidence in the true predictive value of the language centers. Additionally, in clinical practice we do not set a specific threshold for the Edinburgh Handedness Inventory (EHI), the *t*-test score, or the LI. Instead, each of these indices are considered in conjunction with patient factors as described above, given that the clinical utility of each is not in determining with perfect accuracy each area within the language map. Rather, our goal is to create the most sensitive map possible while retaining navigational utility and to relay to our neurosurgeon colleagues how confident we are in the data presented. In our interdisciplinary team, the neurosurgeons understand this also and take the information we present them as a rough roadmap for their interoperative awake mapping, which is considered the gold standard.

### 3.2. Lesion to Activation Distance

**Figure 1:**
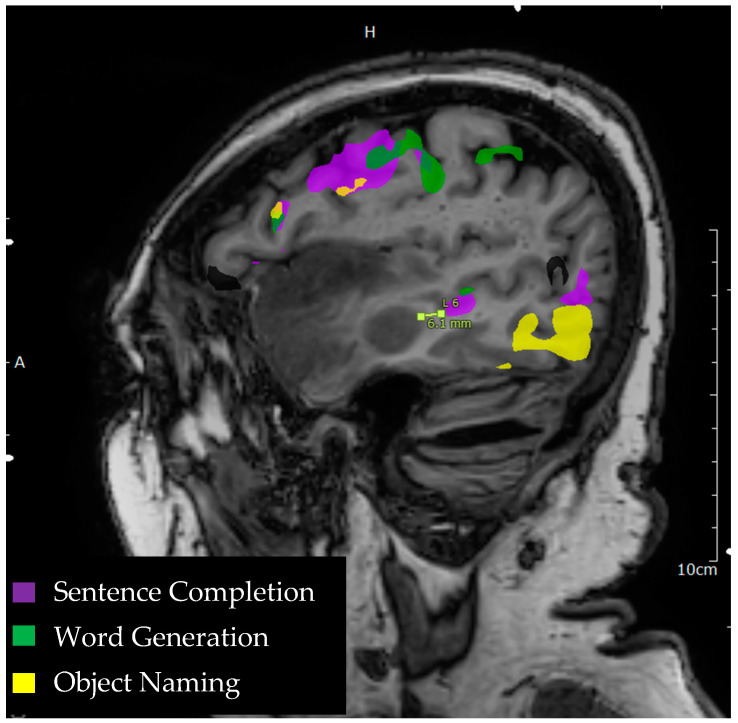
Language tasks eliciting BOLD activation in primary language areas in the left hemisphere. Sagittal view of nodes of BOLD activation for word generation (green), sentence completion (purple), and object naming (yellow) tasks for a single individual. Lesion to activation distance measurement is depicted approximately 6.1 mm from the edge of a sentence completion activation in the middle temporal gyrus to the edge of anterior temporal lobe lesion.

In addition to identifying language laterality, lesion-to-activation distance is another metric provided by preoperative fMRI that predicts postoperative functional outcomes and is useful for guiding intraoperative electrocortical mapping [41,42]. This metric calculates the distance from either the border of the lesion to the center of a functional activation or from the border of the lesion to the border of a node of functional activation (Figure 1). While these distances are useful in surgical planning and predicting postoperative outcomes, it must be noted that allowances must be made for error as these distances are neither exact nor static. BOLD activations are not anatomically precise and activations within a margin of approximately 1 cm from the lesion have been shown to predict postoperative outcomes [43]. In addition, the location of these activations may change relative to the lesion location due to changes in intracranial pressure once the craniotomy is opened or there is a tissue shift as the tumor is debulked. Both the lesion-to-activation distance and LIs are useful in surgical planning and in predicting post-operative deficits. For example, Kundu and colleagues (2013) found that larger tumors are associated with more bilateral activation, especially when the tumor was closer to an area of activation in primary receptive (Wernicke’s area) language areas [44]. Shorter distances between the tumor location and fMRI activations in the primary receptive and primary expressive areas (Wernicke’s and Broca’s areas, respectively) were associated with postoperative aphasia. Additionally, lesions that were closer to Wernicke’s area were associated with greater bilateral activation for Broca’s area, which the authors argue represents a disruption of the overall language network by the encroaching lesion.

Lesion to activation distance has also been shown to be related to preoperative language performance. Riley and colleagues (2022) [45] examined the lesion to activation distance between fMRI activations in Broca’s and Wernicke’s area to the tumor location in relation to the verbal fluency performance. Individuals with lesion to activation distances of less than 10 mm to either Broca’s area or Wernicke’s area had significantly poorer verbal fluency performance than those with lesions greater than 10 mm from these primary language areas. While these studies highlight the utility of fMRI in characterizing postoperative risk, they have largely focused on Broca’s and Wernicke’s areas as the primary areas of interest in characterizing the language network for preoperative planning.

Lesions to Broca’s and Wernicke’s areas are associated with postoperative deficits and should be carefully localized preoperatively and mapped intraoperatively. However, as will be discussed in the next sections, it is essential to consider the integrity of the whole language network especially in light of evidence that significant postoperative language deficits can arise from damage to language areas typically considered ancillary. Furthermore, evidence suggests that we must reconsider whether resection of cortex in these primary language areas is truly associated with the aphasias typically ascribed to these regions.

### 3.3. Broca’s Region

The left inferior frontal gyrus (IFG), comprised of Brodmann areas 44 and 45, gained its moniker Broca’s area from Paul Broca who observed speech production deficits in two historic stroke cases. Subsequently this region of the IFG became synonymous with speech production, despite accumulating evidence to the contrary [46]. Notably, in their recent study detailing long term speech production outcomes in a sample of stroke survivors, Gajardo-Vidal and colleagues (2021) demonstrated that long term speech production impairments were predicted by the degree of damage to the anterior portion of the arcuate fasciculus irrespective of damage to the cortex in Broca’s area [47]. Additionally, evidence from neurosurgical resections suggests that postoperative speech production deficits typically arise from resection of the sensorimotor cortex and supramarginal gyri rather than from Broca’s area and improve post-resection by approximately one month [48]. Broca’s region has been theorized to involve two functionally distinct areas; a language selective region which cooperates in a frontotemporal network subserving syntactic processing, and a domain general region which cooperates in a frontoparietal network involved in working memory, selection, and cognitive control [49]. Its precise role in language remains debated.

**Figure 2:**
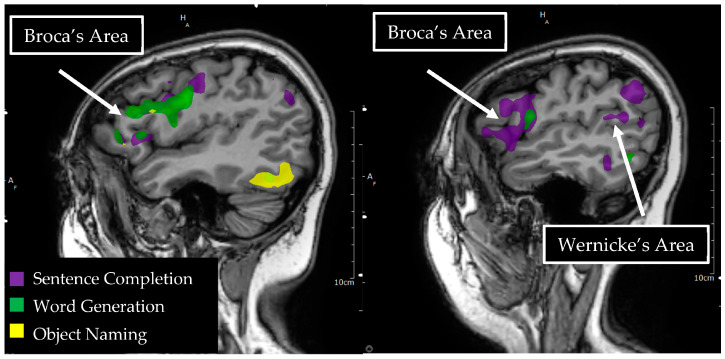
Language tasks eliciting BOLD activation in primary language areas in the left hemisphere. Sagittal view of nodes of BOLD activation for word generation (green), sentence completion (purple), and object naming (yellow) tasks for a single individual. Overlapping nodes of activation are visible for word generation, sentence completion, and object naming in the inferior frontal gyrus, reflecting a functional Broca’s area. A node of activation is visible for sentence completion in the superior temporal gyrus, reflecting a functional Wernicke’s area.

Broca’s area is a functional region with considerable intraindividual differences in its anatomic location and morphology [50], which has made group and atlas based fMRI analyses challenging [51]. An alternative approach has been to use a functional localizer task, rather than defining a region of interest anatomically. Several localizer tasks have been well validated for eliciting activation in one functional area of Broca’s region, including sentences vs. nonwords and speech vs. acoustically degraded speech [52]. The second functional area within Broca’s region appears to be recruited more for tasks which involve greater structural complexity, cognitive control, and increased task difficulty [53,54,55]. In their recent review of paradigms used to elicit language maps for preoperative fMRI for tumor patients, Manan and colleagues (2020) found that that the majority of studies (86%) used a word generation task for localization [56]. Word generation tasks in the reviewed papers included verb, noun, and sentence generation tasks, which elicited consistent activations in the inferior frontal gyrus (Broca’s area and extended IFG) (Figure 2).

With the aid of electrocortical mapping, the pars triangularis and pars opercularis regions of the left inferior frontal gyrus (including anatomical Broca’s area) have been found to be a safe corridor for the resection of insular lesions [57,58]. No long-term postoperative deficits in language or motor function were observed for any of the patients included in these prior studies. In another case series detailing electrocortical mapping and surgical resection of gliomas in the dominant IFG (Broca’s area), the authors found positive language mapping sites in approximately 65% of patients. Only two patients experienced an intraoperative decline in naming during monitoring and thirteen had stable performance throughout monitoring. All patients exhibited a recovery of their language functions compared to the initial presentation after two weeks [59]. Additionally, there is evidence to suggest that functional reorganization of Broca’s area may take place in response to the adjacent glioma. A recent case report detailed a translocation of Broca’s area for a 27 year old patient with a prior resection of the left frontotemporal glioma [60]. fMRI conducted one year postoperatively found significant activation of Wernicke’s area in the left hemisphere and significant activation in the right homologue of Broca’s area in response to word generation and single word semantic decision-making tasks. The authors concluded that this likely represented reorganization of function to the contralateral hemisphere in response to a low-grade glioma. These postoperative findings, along with the aforementioned evidence that Broca’s aphasia may result from damage to the anterior portion of the superior longitudinal fasciculus or cortex in regions typically considered ancillary to language function (supramarginal gyrus), suggest that characterization of the broader language network, rather than preferential attention to Broca’s cortex per se, is essential to preserving language function.

### 3.4. Wernicke’s Region

Wernicke’s region is ill-defined anatomically, consisting of the superior temporal gyrus in Wernicke’s initial account [61], but also including the inferior parietal lobule and the middle temporal gyrus in other accounts. This variability in the definition of Wernicke’s area is likely attributable to wide variability in the precise location of lesions which resulted in the clinical syndrome known as Wernicke’s aphasia [62]. Variation in the anatomic definition of Wernicke’s region has likely resulted in inconsistencies in its proposed role in language processing. In their 2016 review, Ardila, Bernal, and Roselli discuss Wernicke’s area as consisting of a core area in the superior and middle temporal gyri (Brodmann area 21, 22, 41, and 42) and a peripheral area extending into the inferior temporal, posterior middle temporal, temporal pole/anterior temporal, angular, and supramarginal gyri (Brodmann area 20, 37, 38, 39, 40) [63]. The authors argue that the core area is involved in phonological processing and word recognition, with the peripheral area subserving language association and broader semantic processing. Damage to Wernicke’s area is associated with severe aphasia, often consisting of fluent but incomprehensible speech with deficits in auditory short term memory and language comprehension [64].

Due to the severe deficits associated with damage to Wernicke’s area, this functional region has long been considered inoperable in the context of epilepsy or tumor surgery. However, a number of individual case studies have demonstrated successful glioma resections in Wernicke’s area with awake intraoperative mapping with direct electrocortical stimulation. In a case series by Sarubbo and colleagues, intraoperative mapping for tumor resection with direct electrocortical stimulation of superior temporal gyrus and supramarginal gyrus produced anomias and semantic paraphasias [65]. In one case, no language eloquent cortex was identified in the left superior temporal, middle temporal, or supramarginal gyri. This allowed for complete resection of suspected low-grade glioma within these regions. In the other cases in this series, eloquence was identified around tumor margins but with areas identified as safe for transcortical tumor resection within the traditional Wernicke’s area. Positive mapping sites in these cases were also identified once resection reached the level of major subcortical white matter tracts, with semantic paraphasias identified upon direct electrical stimulation of the inferior frontal-occipital fasciculus (IFOF) and phonemic paraphasias upon stimulation of the arcuate fasciculus. No postoperative language deficits were documented in any of the cases in this series. Wernicke’s area has also been documented as a viable transcortical approach for cavernous angioma resection [66]. In another case study by Sarubbo and colleagues, direct electrocortical stimulation of the posterior middle temporal gyrus evoked semantic paraphasias and stimulation of the posterior portion of the superior temporal gyrus evoked anomias. No positive mapping sites were observed in BA22, the posterior superior temporal gyrus, though this region is considered central to the Wernicke’s area (Figure 2). Transcortical resection through this region was successful in removing the subcortical cavernous angioma without significant language deficits postoperatively. This case series again highlights intraindividual variability in the functional organization of primary language regions, allowing for the successful resection of regions typically presumed inoperable.

Preoperative functional neuroimaging is an essential tool providing an initial map of the potential language eloquent cortex, especially within a functional region that is so highly variable in terms of its anatomic location such as Wernicke’s area. Presurgical fMRI paradigms have relied on sentence completion to localize Wernicke’s area in presurgical tumor cases, as sentence completion is more reliable than word generation or category naming in eliciting Wernicke’s activation [67]. In the case of presurgical planning for tumor resection, functional neuroimaging also allows for the characterization of functional regions that may have reorganized in the setting of encroaching tumor tissue. For example, Petrovich and colleagues observed fMRI activation in Wernicke’s right homologue in an individual with a left temporal parietal glioma in the suspected Wernicke’s area [68]. Given that this patient was observed to have left lateralization for Broca’s area and had intact performance on neuropsychological measures of receptive and expressive language, this functional translocation on the fMRI was thought to represent the reorganization of function in the context of a low-grade glioma.

The reorganization of function may also occur in the setting of a prior resective surgery for glioma, allowing for more complete resection later, even when eloquence is identified within the tumor margins. In one case report, a patient underwent an initial awake resection of a left temporal glioma, which was incomplete due to positive language mapping sites during this initial surgery [69]. A second surgery was performed approximately three and a half years later due to tumor growth and increased seizures. During the second awake procedure no positive language mapping sites were observed, allowing for the complete resection of Wernicke’s area in the left hemisphere (posterior superior and middle temporal gyri), suggesting for the reorganization of these previously eloquent sites. Postoperative fMRI demonstrated the recruitment of a distributed bilateral network in the inferior frontal gyrus, supplemental motor area, supramarginal gyrus, superior temporal gyrus, and angular gyrus with the exclusion of resected areas in the left superior temporal and middle temporal gyri. Evidence of functional reorganization in response to an encroaching glioma and post-surgical reorganization after the decortication of these primary language regions emphasizes the importance of intraindividual language mapping as well as the crucial role that ancillary cortical language sites play in plasticity and functional compensation [69,70].

### 3.5. Ancillary Language Centers

The ability to engage in complex daily social and professional functions is an emergent property of numerous discrete cognitive functions and their interactions. More modern connectome models of brain-behavior relationships argue against a modular localizationist model in which brain regions have discrete specialized functions in a one to one map [71]. Attending only to discrete language functions such as object naming or verbal fluency and to the traditional language areas believed to underlie them ignores the richness of human language and may fail to capture the functional impairment that arises from damage to eloquent ancillary language regions and the connections among them [44,72]. We subsequently review evidence of the essential contributions to language functioning of ancillary language areas, as well as impairments arising postoperatively from damage to these regions and fMRI tasks for eliciting BOLD activation in these regions for adequate presurgical mapping.

### 3.6. Dorsolateral Prefrontal Cortex

**Figure 3:**
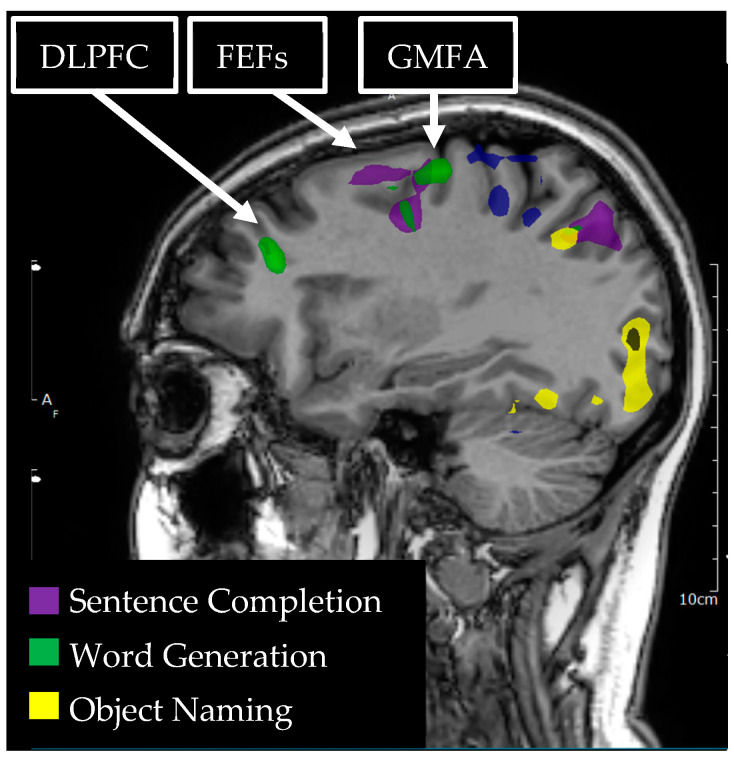
Language tasks eliciting BOLD activation in left hemisphere dorsolateral prefrontal cortex (DLPFC), graphemic motor frontal area (GMFA), and the frontal eye fields (FEFs) for a single individual. Sagittal view of nodes of BOLD activation for word generation (green) and sentence completion (purple) tasks. Nodes of activation are visible for each language task in each of the DLPFC, GMFA, and the FEFs.

The dorsolateral prefrontal cortex (DLPFC) is located in the middle frontal and superior frontal gyri, comprised of the Brodmann areas 46 and 9 (Figure 3). The DLPFC has been traditionally associated with executive control functions including task switching, inhibition, planning, and working memory [73]. The DLPFC is also an important node of language processing, likely contributing to multiple aspects of language function. In their recent review, Hertrich, Dietrich, Blum, and Ackerman (2021) argue that the DLPFC is an important region for sentence processing, creating a coherent narrative, top down speech processing, cooperative conversational exchange, prosody, and language control/switching for bilingual and multilingual people [74]. Transcranial direct current stimulation (tDCS) is a noninvasive alternative to direct electrocortical mapping and is used to create a transient functional lesion. tDCS studies of both language production and comprehension during transient de-activation of DLPFC have elicited slower language comprehension and more production errors during the application of tDCS to the DLPFC, as compared to a sham condition [75].

Intraoperative direct electrocortical stimulation of the DLPFC has elicited working memory disturbance [76], anomias, semantic paraphasias, and phonemic paraphasias during the object naming performance [77,78,79]. Herbet and colleagues (2018) argue that the DLPFC is an essential hub for semantic processing [80]. The authors observed semantic paraphasias and errors in amodal semantic processing for the direct electrocortical stimulation of sites in both the left and right DLPFC during intraoperative mapping. Evidence from postoperative neuropsychological testing also suggests a role for the DLPFC in semantic processing. In one case study, a patient with a left frontal glioma (WHO grade III oligoastrocytoma) underwent resective surgery with intraoperative neurocognitive testing [81]. Preoperative neuropsychological testing did not demonstrate any impairments in executive or language functions. Intraoperative monitoring observed verbal fluency and naming difficulties during the resection of areas of infiltration surrounding the core of the lesion, resulting in cessation of the resection, however approximately 90% of the tumor was removed according to postoperative imaging. Postoperative neuropsychological testing demonstrated deficits in auditory working memory, executive set shifting, and verbal fluency. She evidenced a particular impairment in semantic processing with difficulties accessing conceptual information, generating semantically related words, and frequent semantic paraphasias. Given the resection cavity depicted in this case, it is possible this long-term deficit is a result of damage to major white matter tracts, including the superior longitudinal fasciculus (SLF) and IFOF which are positioned below the DLPFC in this region.

Functional MRI studies of the DLPFC contributions to language function have largely investigated its shared role in executive functioning/working memory and language control processes, especially for bilingual and multilingual individuals. Meta-analytic evidence suggests that the DLPFC cooperates with a network of other regions including the pre-supplementary motor area, the anterior cingulate cortex, and the precuneus during language switching for bilingual individuals [82]. DLPFC activation has also been observed processing complex syntax [83] and in reading comprehension [84], especially when working memory demands were higher in the task employed. Clinically, fMRI tasks used to elicit language activation in the DLPFC for presurgical planning have included sentence completion [85], word generation [86], verb generation [87], and rhyming [88].

### 3.7. Graphemic Motor Frontal Area (Exner’s)

The Graphemic Motor Frontal Area (GMFA) or Exner’s Area sits posterior to the DLPFC in the middle frontal gyrus at the most inferior part of Brodmann area 6 (Figure 3). It is believed to subserve the conversion of orthographic representations into motor programs specific to the written expression of language, and cooperates with a network of other regions that are also involved in written language, including the parietal cortex and fusiform gyrus [89]. Strokes in GMFA have produced impairments in writing and in the transcription of dictated words with intact speech, in verbal comprehension, and in the ability to copy written text [90]. This dissociation of the motoric act of writing and language comprehension/expression from the ability to write words spontaneously or from dictation implicates GMFA in the conversion of phoneme to grapheme.

Intraoperative mapping and postoperative cognitive performance data from patients who have undergone surgery has been more equivocal about the precise role of GMFA, potentially due to intraindividual differences in functional anatomy and the location of the tumor. Direct electrocortical stimulation of GMFA has produced anarthria [91], anomia [78], and disruptions in handwriting without disruption of hand motor control or oral language performance [92]. Scarone and colleagues (2009) detailed 15 case studies of patients who underwent awake craniotomy for glioma resection and subsequently experienced deficits in written language production [93]. The authors reported deficits in writing following the resection of a glioma in area BA6, which encompasses both the GMFA and the more central supplementary motor area (SMA), consisting of slow, irregular writing with numerous letter substitutions. Notably, these cases did not have any symptoms consistent with a motor SMA syndrome. Deficits in handwriting were also reported for cases with lesions spanning the GMFA and into the inferior frontal gyrus. For these patients, postoperative deficits in handwriting consisted of written semantic paraphasias, graphemic paraphasias (written phonemic paraphasias). Preoperative fMRI was not performed in these cases to identify the functional region of GMFA. Of the cases detailed in this study, only those with resections in SMA spanning into GMFA had persistent deficits in handwriting 6 months postoperatively. Though differences in lesion location with overlap into the GMFA, and surrounding inferior frontal and SMA regions, make it difficult to infer the precise deficit that would result from the resection limited solely to the GMFA.

Tasks used to elicit fMRI activation in GMFA have included sentence completion, letter strings, object naming, verb generation, naming from written description, and naming from aural description [85,94,95,96]. Benjamin and colleagues (2017) developed a protocol designed for clinical application in presurgical planning consisting of three language tasks that reliably elicit activation in six major language areas, including the GMFA. These tasks consisted of visual object naming, naming from aural description, and naming from written description.

### 3.8. Frontal Eye Fields

The Frontal Eye Fields (FEFs) are situated just posterior to the DLPFC in the superior frontal gyrus and are generally thought to be comprised of the Brodmann area 8 (Figure 3). However, there is significant anatomic variability in the location of the functional region of the FEFs, when applying direct electrocortical stimulation during the intraoperative mapping for tumor resection. Pallud and colleagues (2018) found that direct electrocortical stimulation of FEFs elicited involuntary saccadic eye movements in areas spanning Brodmann areas 6, 8, and 9 [97]. The FEFs have been shown to be involved in top-down attentional allocation and eye movements (for review see Vernet et al., 2014) [98]. A number of language tasks result in fMRI activation in FEFs, including reading comprehension [99] and sentence completion [85], potentially due to the demands on automatic saccadic eye movement from reading tasks [100,101]. While it is possible that FEFs may not be directly involved in core language function per se, it serves essential supportive functions of attentional allocation for language processing, including reading and sound orienting/localization [102].

To our knowledge, no reports have detailed postoperative outcomes for tumor resection of language dominant FEFs. In one case report of preoperative fMRI and awake resection of low-grade glioma in right FEFs, self-paced symmetric horizontal saccades were used to map the activation of FEFs on fMRI [103]. Presurgical fMRI identified significant activation in the left FEFs, contralateral to the lesion in the right FEFs. Intraoperative mapping of the lesion using direct electrocortical stimulation produced positive mapping sites with the induction of contralateral smooth eye movements and saccadic suppression upon interrogation of right FEFs. Postoperative outcomes were not reported in this case study. Preliminary reports have noted consistency between presurgical fMRI and intraoperative positive mapping sites for FEFs, though to our knowledge no systematic examination of this concordance has been reported in the literature [103,104]. Additional investigation of the contributions of the FEFs to language functioning and of the utility of presurgical fMRI to presurgical planning for the resection of tumors within FEFs remains necessary.

### 3.9. Pre-SMA/Language SMA

**Figure 4:**
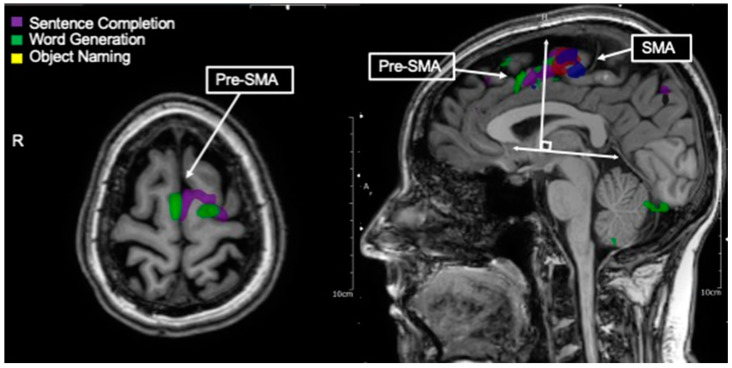
Language tasks eliciting BOLD activation in pre-supplementary motor area (pre-SMA also called language SMA). Axial view of nodes of BOLD activation for word generation (green), and sentence completion (purple) tasks for single individual. Overlapping nodes of activation are visible in the pre-SMA/language SMA. Depicted in the right-handed figure, language tasks eliciting BOLD activation in pre-SMA as destinguished from hand movement (blue) and lip movement (red) activations in SMA. These nodes may generally be distinguished by a line drawn perpendicular from the AC-PC line at the anterior commissure.

Just anterior to the supplementary motor area (SMA; medial BA 6) is a functional region termed Pre-SMA or language SMA, typically defined as the medial aspect of Brodmann area 8 (Figure 4)and the anterior and medial aspect of Brodmann area 6 [105,106]. Pre-SMA or language SMA may be delineated anatomically from motor SMA using a line drawn perpendicular to the AC-PC line at the anterior commissure [107]. While this area was previously believed to subserve motor planning primarily via the resolution of competing responses [108], more recent literature suggests a prominent role as part of the language network. Lesional studies have demonstrated deficits in speech initiation and articulation in the absence of other language or motor deficits for individuals with stroke in the left language SMA or surgical resection of the left language SMA [109,110,111].

Intraoperative direct electrocortical stimulation of the language SMA during awake tumor resection has resulted in speech arrest [91,112]. In one case study, a preoperative fMRI identified a pre-SMA using phonemic and category fluency tasks abutting a glioma in the superior frontal gyrus [113]. Postoperatively, the patient was unable to initiate speech, but language comprehension and motor initiation were intact, consistent with a pre-SMA syndrome. Postoperative fMRI conducted at three timepoints (12 days, 3 months, and 4.5 months) demonstrated initial increased activation in the right pre-SMA as compared to preoperative fMRI, with no activation in the left pre-SMA. Subsequently, activation was observed again in the remaining posterior region of left pre-SMA at 3 and 4.5 months postoperatively. The timing of this change in activation on the fMRI corresponded to the patient’s recovery of language initiation to near baseline performance and was therefore posited to represent a functional reorganization. Similarly, other studies suggest that language deficits may be transient in the case of lesions to a unilateral SMA, potentially due to the capacity for reorganization of function to the contralateral hemisphere [114]. However, other case studies have reported persistent postoperative deficits in verbal fluency and aspects of executive functioning with language SMA resection [115]. These more persistent deficits may be attributable to damage to deep white matter tracts connecting the language SMA to other cortical areas within the language network, as resection in this case included anterior aspects of the SLF. As mentioned previously during the discussion of Broca’s area, damage to the connections among language eloquent cortical regions may result in more longstanding language deficits and make reorganization of function less likely. This highlights the importance of considering the integrity of the language network as a whole for functional preservation, rather than prioritizing discrete cortical regions. Language SMA has crucial connections to other cortical regions in the language network, including to the inferior frontal gyrus/Broca’s area through the frontal aslant tract [116,117,118]. Direct electrocortical stimulation of language SMA and subcortical white matter below language SMA in the presumed frontal aslant during intraoperative mapping for tumor resection has resulted in speech arrest [118,119]. In addition, damage to the frontal aslant has produced a language SMA syndrome in the absence of damage to the language SMA cortex [120].

Functional MRI activation in the language SMA has been elicited by semantic decision making [121], language switching in bilingual individuals [122], sentence completion, word repetition, and other tasks involving response selection and language production (for review see Hertrich et al., 2016) [123]. For preoperative mapping of the language SMA, activation in this region has been reliably elicited by verb generation, phonemic fluency, and category fluency [124,125]. Lesions in the unilateral SMA were associated with greater fMRI activation on the contralateral side, as well as reduced fMRI activation on the side of the lesion, potentially reflecting a reorganization of function or disruption of the local BOLD signal by the encroaching lesion. Resection of language SMA activations identified on preoperative fMRI has been associated with language SMA syndrome postoperatively, suggesting that fMRI can reliably predict postoperative outcomes and identify areas of functional eloquence preoperatively [125]. Other studies of preoperative fMRI which predict outcomes after resection of lesions in language SMA have not found an effect of the lesion in accordance to the activation distance [126,127]. The authors argue that the use of preoperative fMRI and the identification of language activations near the lesion may have resulted in more conservative surgical approaches, allowing for the preservation of language function and therefore restricting the range of outcomes for individuals with differing lesion to activation differences, making the detection of a statistical effect untenable [126].

### 3.10. Inferior Parietal Lobule/Geschwind’s Area

**Figure 5:**
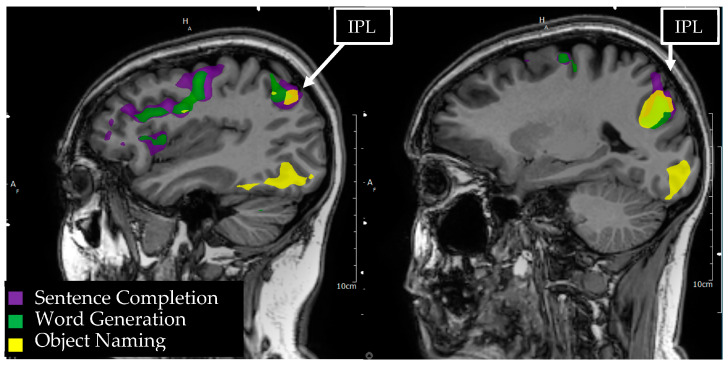
Language tasks eliciting BOLD activation in the inferior parietal lobule (IPL) for a single individual. Sagittal view of the left hemisphere depicts nodes of BOLD activation for object naming (yellow), word generation (green), and sentence comprehension (purple) tasks. Overlapping nodes of activation for object naming and sentence comprehension are visible in the angular and supramarginal gyri of the IPL.

The inferior parietal lobule (IPL), often called Geschwind’s Area, sits just inferior to the postcentral gyrus and is inferior and lateral to the superior parietal lobule as divided by the intraparietal sulcus [128]. It is composed of the angular gyrus and supramarginal gyrus (Brodmann’s Area 39 and 40) (Figure 5). Lesional studies have demonstrated a role for the dominant IPL in semantic knowledge, particularly with respect to semantic knowledge pertaining to the behavioral features of objects and how they interact, as well as in predicting events and actions [129]. The dominant IPL also plays an important role in phonetic processing. Damage to the dominant IPL cortex resulting from a stroke is associated with impairments in speech repetition, irrespective of damage to the underlying arcuate fasciculus [130]. Neuroimaging studies of phonetic processing also found significant activations in the inferior parietal lobule during the discrimination of highly overlapping phonetic speech sounds [131] Strokes to the dominant IPL have also resulted in alexia and agraphia [132].

Direct electrocortical stimulation of the IPL has produced alexia, anomia, semantic paraphasias, and phonemic paraphasias during word reading and object naming tasks during awake glioma resection [133]. In this case series, postoperative language deficits including alexia, anomia, word finding difficulties, impaired repetition, and word finding difficulties were observed after the resection of IPL glioma. Patients were more likely to experience postoperative language deficits if intraoperative positive language mapping sites were identified on electrocortical stimulation within the resection area, highlighting the importance of intraoperative mapping for the prediction of outcomes. Older adults were also more likely to experience long term language deficits, potentially confounded by the higher rate of high-grade gliomas in this subset of the study population. In another study of awake resections of IPL gliomas, direct electrocortical stimulation produced anomia, semantic paraphasias, and phonetic errors upon interrogation of the supramarginal gyrus and angular gyrus within the IPL [134]. In this case series, subcortical electrical interrogation of white matter tracts underneath the IPL cortex resulted in phonemic errors most commonly, but was also observed to produce semantic paraphasias and syntax errors. Resection was paused at all positive cortical and subcortical mapping sites and patients experienced only transient language deficits that persisted no more than two weeks post-surgically.

Presurgical fMRI paradigms used to identify language eloquent cortex prior to the resection of gliomas in the IPL have included naming, verb generation, phonemic fluency, and rhyming [29,135,136]. Roux and colleagues (2003) documented correlations among presurgical fMRI activations and intraoperative direct electrocortical stimulation findings [136]. The authors observed activations in the angular gyrus and supramarginal gyrus for both naming and verb generation tasks, with only marginal prediction of positive intraoperative mapping findings for each task alone (sensitivity 22–36%, *p* < 0.005). When using activations for both tasks together, along with their overlap, prediction of positive intraoperative mapping sites improved substantially (sensitivity 59%, *p* < 0.005). The prediction of positive mapping sites also varied by the threshold chosen, with a less restrictive threshold resulting in a higher sensitivity when considering both tasks combined (66%, *p* < 0.05). The authors emphasize that while this study highlights the predictive utility of fMRI, it also shows the necessity of direct electrocortical stimulation mapping, as fMRI alone cannot identify language eloquent cortex with a sufficient enough sensitivity to avoid postoperative deficits.

### 3.11. Basal Temporal Language Area

The basal temporal language area (BTLA) refers to a functional region within the posterior and inferior temporal lobe that has been implicated in language function since at least the late 1800′s [96]. While there is some disagreement about which specific cortical structures are functionally involved in the BTLA, it is generally thought to be comprised of the posterior aspects of the fusiform, parahippocampal, and inferior temporal gyri. Luders and colleagues (1989) described a significant aphasia produced in a subset of patients upon electrical stimulation at a high intensity of the language dominant basal temporal region, particularly the fusiform gyrus [137]. The language disruption observed in these patients involved both receptive and expressive language abilities, and they noted that seizure onset in this zone was characterized by early speech arrest [137,138,139]. Interestingly, stimulation at a lower intensity in this region was associated with object naming deficits only.

Initial studies of the surgical resection of this region suggested no significant impairment arising from its surgical removal, though more recent studies suggest more significant functional consequences. Pouratian et al. (2003) reported a case involving the surgical resection of the dominant fusiform gyrus in a patient receiving preoperative fMRI, pre- and post-operative neuropsychological evaluation, and intraoperative functional mapping [140]. fMRI activations suggested a possible language eloquent cortex within that region, which was confirmed intraoperatively with direct cortical electrostimulation. Notably, stimulation appeared to produce a modality specific deficit in naming, but only for living objects. A statistically significant increase in the error rate for naming of living vs. nonliving objects was also observed on postoperative neuropsychological testing, suggesting the possibility of a modality specific organization for the retrieval of object names in the BTLA [140]. Similarly, stimulation of the BTLA was investigated in a small sample of Japanese speaking patients [141]. Japanese is characterized by two parallel systems of writing, one involving phonetic representations that form whole words and another using individual symbols for whole word representations. Moreover, these symbols are often closely associated with the objects they represent in Japanese culture. Stimulation of the region resulted in an inability to name visually presented objects as well as symbolic whole word representations, while reading of individual phonetic symbols was unaffected. Auditory presentation of these was also unaffected, suggesting a dedicated role for the BTLA in binding visual semantic information with phonological representations of semantic concepts, with a separate processing stream likely involved in visual phonological processing [141]. Additional support for the BTLA’s crucial role in language processing comes from studies of functional connectivity, which have revealed bidirectional connectivity between the BTLA and primary receptive language regions (i.e., Wernicke’s area).

**Figure 6:**
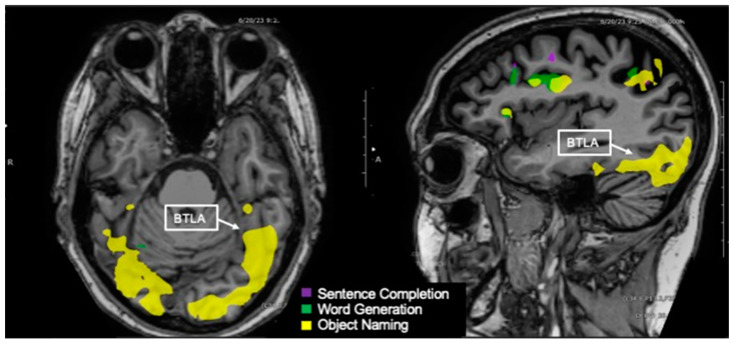
Language tasks eliciting BOLD activation in basal temporal language area (BTLA) for a single individual. Axial view (**left**) and sagittal view (**right**) of nodes of BOLD activation for the object naming (yellow) task. The sagittal view shows the left hemisphere. Nodes of activation for object naming are visible in the BTLA.

The fMRI task most commonly used to elicit BTLA activations (Figure 6) is some variation of the visual object naming task, though other language tasks may also generate BTLA activations to a lesser extent. Direct comparisons of preoperative fMRI to intraoperative direct cortical electrostimulation have revealed a strong sensitivity, though lower specificity, in the localization of language eloquent sites [86] with a combined task protocol of object naming, verb generation, and sentence comprehension, evidencing 100% sensitivity though only 61% specificity when compared to electrostimulation. However, object naming is generally considered less effective in lateralizing hemispheric language dominance despite its good functional localization, and therefore, it is most commonly used as only one in a series of preoperative language mapping fMRI tasks [86].

## 4. Conclusions

In the era of fMRI, one now has the chance to help preserve critical language functions during brain surgery for tumors. Our review of preoperative neuroimaging and postoperative language outcomes for surgical resection in eight nodes of the language system shows that significant language deficits can arise from damage to regions not circumscribed to traditional Broca’s and Wernicke’s areas. By knowing these nodes when performing fMRI, one can gain confidence in knowing what a patient’s true laterality is. Furthermore, this review highlights the importance of presurgical mapping of language areas traditionally presumed secondary or ancillary. fMRI has reasonable concordance with the direct intraoperative electrocortical mapping in each of these regions and remains a useful clinical tool for communicating informed consent about the functional risk to the patient, for planning the surgical approach, and for the selection of intraoperative mapping tasks. Additionally, while fMRI should not be used in isolation with the available direct awake electrocortical stimulation mapping of the language eloquent cortex, it provides adequate functional mapping for avoiding postoperative functional deficits when awake methods are not feasible [142].

Furthermore, the integrity of the language network as a whole must be considered for presurgical planning, including the essential language white matter tracts that connect among language eloquent cortical regions. As discussed previously, a number of studies have demonstrated language deficits resulting from the disconnection of cortical regions due to damage to underlying white matter tracts, even if language eloquent cortical areas are spared [47,81,120]. Indeed, preoperative white matter integrity within major language tracts is a predictor of postoperative recovery from aphasia [143]. These findings stress the importance of increasing attention toward the white matter tracts connecting these regions and to the integrity of language networks more broadly for the prediction of postoperative outcomes.

Task based fMRI that focuses primarily on identifying nodes of activity remains the most extensively validated method for presurgical language mapping. This method does not characterize the integrity of the language network as a whole, per se. This review has focused primarily on the importance of characterizing ancillary language regions with the understanding that these regions contribute substantially to the language network. Though it has not been within the scope of this review to discuss metrics of quantifying the integrity of the language network itself, these metrics are an important future direction of research for clinical use. Briefly, functional connectivity analyses, both through task based and resting state fMRI, are methods of measuring the functional cooperation among networks of eloquent regions. Gliomas have been shown to be associated with disruption to both local and broad functional networks [144]. Decreased language network integrity as defined by the resting state functional connectivity is a significant predictor of language deficits, particularly for individuals with high grade gliomas [145]. Furthermore, pre-and post-operative resting state connectivity analyses have been used to assess changes to attentional and language networks associated with glioma resection [146]. Resting state and task based connectivity analyses are also useful in investigating network changes that occur as a result of dynamic simultaneous processes such as tumor infiltration, the rate of tumor growth, functional reorganization, and functional compensation [147]. Future research is necessary to validate resting state fMRI as well as both task based and resting state network connectivity analyses for clinical use, though several studies have demonstrated promising initial results, cross validating resting state fMRI, task based fMRI, and intraoperative electrocortical mapping for individuals with glioma [148,149].

## Data Availability

Not applicable, this review did not report any data.
